# Supplementary material for: Understanding Drug Resistance of Wild-Type and L38HL Insertion Mutant of HIV-1 C Protease to Saquinavir
Source: Genes (Basel). 2023 Feb 20;14(2):533. doi: 10.3390/genes14020533 (PMC9957153; doi:10.3390/genes14020533)
Supplement: Supplementary file 1 [file genes-14-00533-s001.zip › genes-2216235-supplementary.pdf]

Supplementary Information

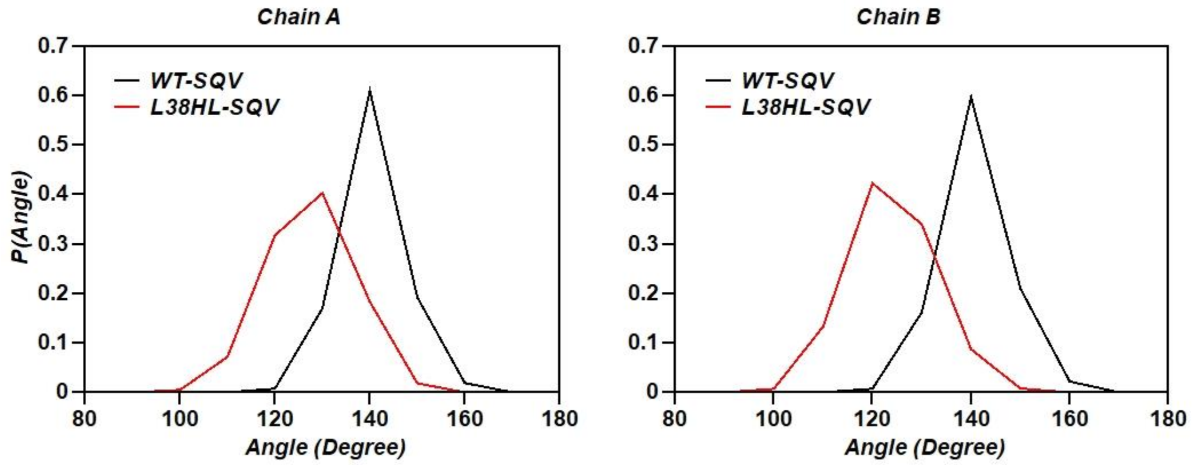

Figure S1: Frequency distribution of Hinge Cα Angles

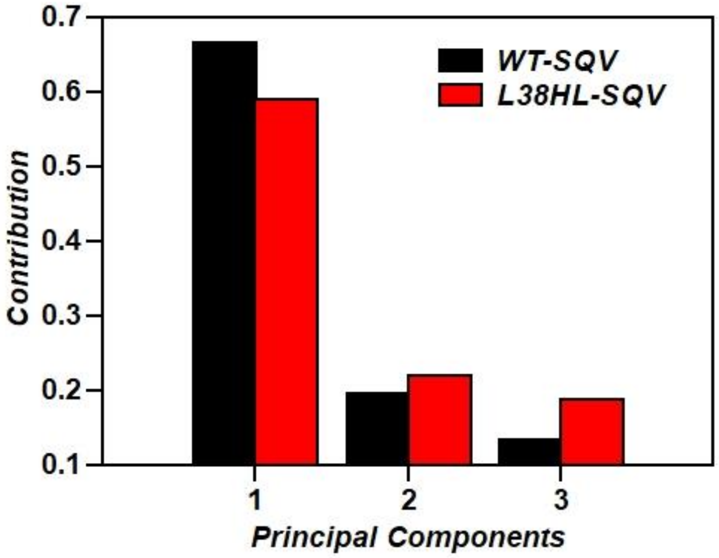

Figure S2: Contributions from the first 3 principal components

Supplementary Table S1

List of hydrogen bonds formed between the homodimer in wild-type

Acceptor	Donor	Occupancy
PRO1@O	PHE198@N	0.3605
GLN2@OE1	THR195@OG1	0.1318
LEU3@O	LEU196@AN	0.460
LEU5@O	ARG186@NH1	0.589
THR26@OG1	THR125@OG1	0.430
ASP29@OD1	ARG107@NE	0.300
ASP29@OD1	ARG107@NH2	0.389
ASP29@OD2	ARG107@NE	0.276
ASP29@OD2	ARG107@NH2	0.365
THR96@O	ASN197@N	0.700
THR96@OG1	ASN197@ND2	0.123
THR96@OG1	GLN101@NE2	0.120
LEU97@O	ILE102@N	0.808
ASN98@O	THR195@N	0.360
PHE99@O	PRO100@N	0.293
PHE99@OXT	PRO100@N	0.339
PRO100@O	PHE99@N	0.353
GLN101@OE1	THR96@N	0.165
ILE102@O	LEU97@N	0.339
LEU104@O	ARG87@NH1	0.728
LEU123@O	THR26@OG1	0.174
GLY148@O	GLY51@N	0.210
THR195@O	ASN98@N	0.717
THR195@OG1	ASN98@ND2	0.107
THR195@OG1	GLN2@NE2	0.118
LEU196@O	ILE3@N	0.787
ASN197@O	THR96@N	0.326
PHE198@O	PRO1@N	0.298
PHE198@OXT	PRO1@N	0.324

Supplementary Table S2

List of hydrogen bonds formed between the homodimer in L38HL

Acceptor	Donor	Occupancy
PRO1@O	PHE202@N	0.437
ILE3@O	LEU200@N	0.416
LEU5@O	ARG190@NH1	0.815
LEU24@O	THR127@OG1	0.523
THR98@O	ASN201@N	0.756
LEU99@O	ILE104@N	0.810
ASN100@O	THR199@N	0.123
PHE101@O	PRO102N	0.36
PHE101@OXT	PRO102@N	0.338
PRO102@O	PHE101@N	0.433
GLN103@OE1	THE98@OG1	0.328
ILE104@O	LEU99@N	0.471
LEU106@O	ARG89@NH1	0.468
ASP130@OD1	ARG8@NE	0.324
ASP130@OD1	ARG8@NH2	0.407
ASP130@OD2	ARG8@NE	0.322
ASP130@OD2	ARG8@NH2	0.462
GLY154@O	GLY53@N	0.122
THR199@O	ASN100@N	0.7941
LEU200@O	ILE3@N	0.8218
ASN201@O	THE98@N	0.3781
PHE202@O	PRO1@N	0.29
PHE202@OXT	PRO1@N	0.315
